# Bone substitutes in orthopaedic surgery: from basic science to clinical practice

**DOI:** 10.1007/s10856-014-5240-2

**Published:** 2014-05-28

**Authors:** V. Campana, G. Milano, E. Pagano, M. Barba, C. Cicione, G. Salonna, W. Lattanzi, G. Logroscino

**Affiliations:** 1 Department of Orthopaedics and Traumatology, Università Cattolica del Sacro Cuore, L.go F. Vito 1, 00168 Rome, Italy; 2 Institute of Anatomy and Cell Biology, Università Cattolica del Sacro Cuore, Rome, Italy; 3 Latium Musculoskeletal Tissue Bank, Rome, Italy

## Abstract

Bone substitutes are being increasingly used in surgery as over two millions bone grafting procedures are performed worldwide per year. Autografts still represent the gold standard for bone substitution, though the morbidity and the inherent limited availability are the main limitations. Allografts, i.e. banked bone, are osteoconductive and weakly osteoinductive, though there are still concerns about the residual infective risks, costs and donor availability issues. As an alternative, xenograft substitutes are cheap, but their use provided contrasting results, so far. Ceramic-based synthetic bone substitutes are alternatively based on hydroxyapatite (HA) and tricalcium phosphates, and are widely used in the clinical practice. Indeed, despite being completely resorbable and weaker than cortical bone, they have exhaustively proved to be effective. Biomimetic HAs are the evolution of traditional HA and contains ions (carbonates, Si, Sr, Fl, Mg) that mimic natural HA (biomimetic HA). Injectable cements represent another evolution, enabling mininvasive techniques. Bone morphogenetic proteins (namely BMP2 and 7) are the only bone inducing growth factors approved for human use in spine surgery and for the treatment of tibial nonunion. Demineralized bone matrix and platelet rich plasma did not prove to be effective and their use as bone substitutes remains controversial. Experimental cell-based approaches are considered the best suitable emerging strategies in several regenerative medicine application, including bone regeneration. In some cases, cells have been used as bioactive vehicles delivering osteoinductive genes locally to achieve bone regeneration. In particular, mesenchymal stem cells have been widely exploited for this purpose, being multipotent cells capable of efficient osteogenic potential. Here we intend to review and update the alternative available techniques used for bone fusion, along with some hints on the advancements achieved through the experimental research in this field.

## Introduction

Bone substitutes are being increasingly used especially in oncologic surgery, traumatology, revision prosthetic surgery and spine surgery [1]. Bone grafting frequency is indeed the second most frequent tissue transplantation worldwide, coming right after blood transfusion. Over two millions bone grafting procedures are performed every year, with more than 500,000 implanted in the US alone [2–5]. This is due to their ease use and handling, improved safety profiles, intraoperative cost and time advantages, and adaptability to a variety of clinical challenges.

The incorporation of a bone graft is defined as the “process of envelopment and interdigitation of the donor bone tissue with new bone deposited by the recipient” [6]. This process follows a typical multistep cascade: initially, the bone graft produces a response leading to the accumulation of inflammatory cells, followed by the chemotaxis of host mesenchymal cells to the graft site. Thereafter, the primitive host cells differentiate into chondroblasts and osteoblasts, a process under the influence of various osteoinductive factors. The additional processes of bone graft revascularization and necrotic graft resorption occur concurrently. Finally, bone production from the osteoblasts onto the graft’s three-dimensional framework occurs, followed by bone remodeling in response to mechanical stress [7, 8].

A bone substitute can be defined as “a synthetic, inorganic or biologically organic combination which can be inserted for the treatment of a bone defect instead of autogenous or allogenous bone” [9]. A wide variety of bone substitutes have been employed over the past 50 years. Bone substitutes can be broadly categorized into bone grafts (autograft, allograft, xenograft), ceramics (hydroxyapatite, TCP, calcium sulphate) and growth factors (DBM, PRP, BMP’S) [10]. The ideal bone substitute should be biocompatible and not evoke any adverse inflammatory response. It should be easily molded into the bone defect within a short setting time. It should be osteoconductive, osteoinductive [11] and resorbable. It should be possibly traceable in vivo; to this aim radiolucency is ideal to allow optimal radiographic assessment. Also, the ideal bone substitute should be thermally nonconductive, sterilizable, and readily available at a reasonable cost [12].

Although autologous bone grafting is still considered the “gold standard” in bony defect repair, the past century has seen significant advances in the development of valid alternatives to natural bone.

The latter half of the twentieth century has seen the evolution of the hydroxyapatite and calcium phosphate-based cements and ceramics. Current advances are being made with the development of tissue-engineered products, incorporating growth factors and stem cells.

Depending on the type of surgery and on the bone loss, many options are possible. Cortical strut grafts are used were mechanic strength is needed. Spongy bone, often morcelized, is more usefull to fill cavitary bone defects or in spine fusion. Ceramics under granule, blocks, or moldable paste shape are normally used to enhance bone heal, but have poor mechanical qualities and stable hardware fixation is necessary. Growth factors are on the market as moldable paste. BMPs are currently indicated only in tibial non union (BMP-7) and spine surgery (BMP-2).

In this report we will review some of the most important biomaterials in each of these categories.

## Bone grafts

### Autografts

**Fig. 1:**
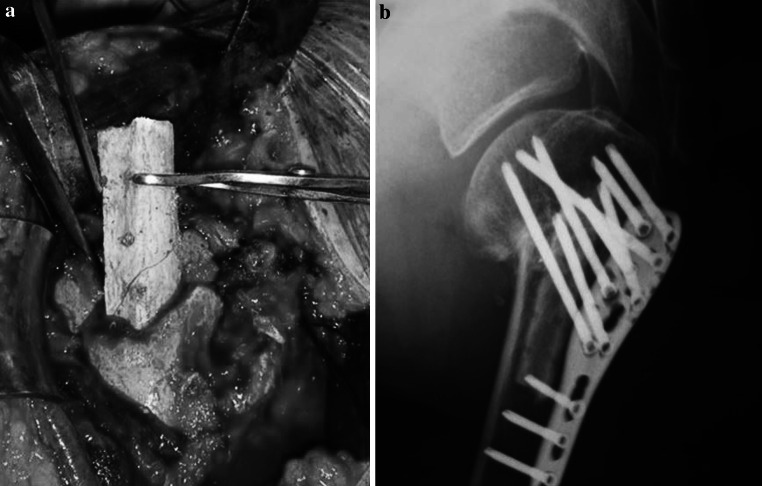
**a** Cortical strut autograft from fibula in a proximal humeral non union treated by ORIF. **b** One year X-ray control show bone healing and the persistence of the autograft

Currently autografts are the “gold standard” in bone substitution [13, 14]. Autologous (or autogenous) bone grafting involves utilizing bone obtained from the same individual receiving the graft [15]. Bone can be harvested from non-essential bones, such as the iliac crest or the fibula (Fig. 1), the chin, the ribs, the mandible and even parts of the skull. Autogenous bone possesses all the properties essential for bone formation: it is osteoconductive and osteoinductive, and it houses growth factors and osteogenic cells with no associated immune or infective-related risks. Autologous bone fracts are slowly replaced by newly formed host bone. The disadvantages of autografts reside in the inherent morbidity: (1) a surgical donor site is required, leading to possible post-operative pain and complications. [16, 17]; (2) a likelihood of blood loss or hematomas, infection, fracture, neurovascular injury, as well as cosmetic deformity, at the explantation site and longer operative time.

Also, autogenous bone availability in a patient represents a significant limit, especially in pediatric patients and in the elderly. An autograft may also be performed without a solid bony structure, for example using bone reamed from the anterior superior iliac spine. In this case there is an osteoinductive and osteogenic action, however there is no mechanical support action, as there is no solid bony structure [18].

### Allografts

**Fig. 2:**
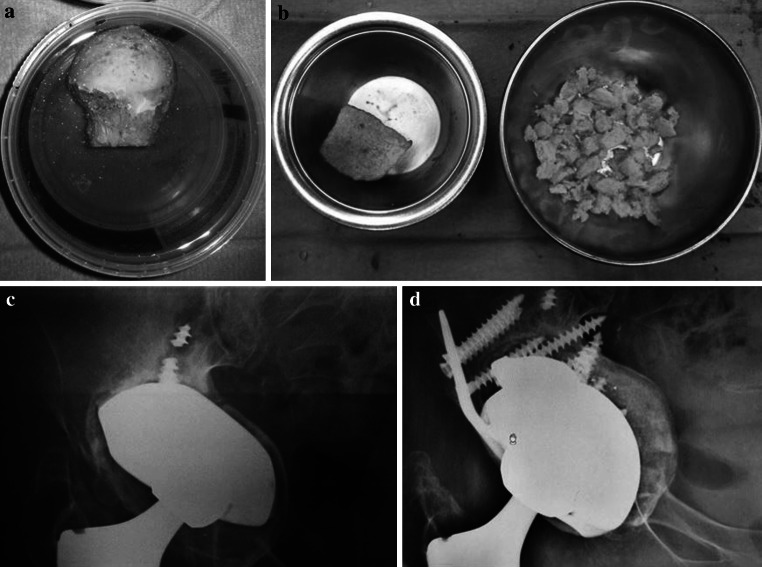
**a**, **b** Morcelized homologous bone graft obtained from a banked femoral head. **c** Severe acetabular bone loss in a mobilized hip revision cup. **d** X-ray control at 2 years with evidence of bony stable osseointegration of the new cup in the remodeled bone graft

Allograft biobanked bone represents a suitable alternative to autogenous bone, being derived from humans as well. Allograft bone can be collected from either living donors (patients total hip replacement surgery) or nonliving donors and must be processed within a bone tissue bank (Fig. 2). Bone Tissue Banks fastly grew up since the’80 years but doubts and concerns arise about costs and problems related to storing [19].

Donor bone is osteoconductive, weakly osteoinductive (growth factors may still be present, depending on the processing). Also, allografts often require sterilization (gamma-irradiation), with detrimental effects on mechanical properties of bone, and deactivation of proteins normally found in healthy bone. Concerns on the potential infective risks were raised, though since 1989 only 2 documented cases of HIV were reported with a risk rate of 1:1.6 milions [20]. Anyway, current procedure for donor bone procurement and processing are designed to significantly limit the possible transmission of knonw pathogens [21].

Other more important infective risk were reported on HBV (1 case), HCV (2 cases), one fatal infection by Clostridium Difficilis, and 26 bacterial infections [22–24].

The limits of such transplants are costs, laborious procedure (tissue processing, harvesting), mechanical resistance (in freeze dried and irradiated), limited osteoinduction and risk of infection.

### Xenografts

**Fig. 3:**
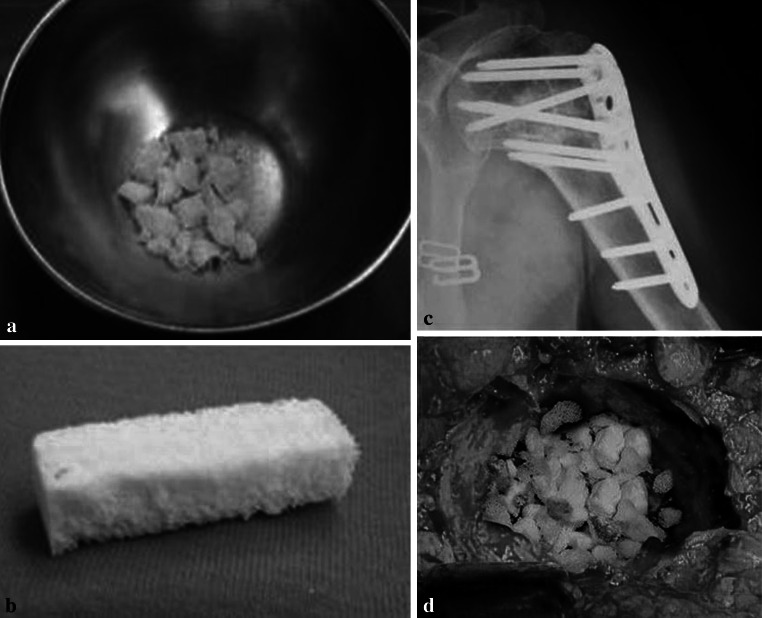
**a**, **b** Bovine bone substitute (Xenograft) in chips and blocks shape. **c** The xenograft is clearly visible and not resorbed in a well bone healed proximal humeral fracture at 1 year of follow up. **d** Acetabular bone defect filled with the same material

Xenograft bone substitutes have their origin from a species other than human, such as bovine bone (or porcine bone) which can be freeze dried or demineralized and deproteinized (Fig. 3). Bovine bone was first introduced by Maatz and Bauermeister in 1957 [25]. Xenografts are usually only distributed as a calcified matrix. Madrepore and or millepore type of corals are harvested and treated to become “coral derived granules” (CDG) and other types of coralline xenografts [26]. Coral based xenografts are mainly calcium carbonate (and an important proportion of fluorides, useful in the context of grafting to promote bone development) while natural human bone is made of hydroxyapatite along with calcium phosphate and carbonate. The coral material is thus either transformed industrially into hydroxyapatite through a hydrothermal process, yielding to a non-resorbable xenograft, or simply the process is omitted and the coralline material remains in its calcium carbonate state for better resorption of the graft by the natural bone. The coral xenograft is then saturated with growth enhancing gels and solutions [27]. In January 2010 Italian scientists announced a breakthrough in the use of wood as a bone substitute, though this technique is not expected to be used for humans until at the earliest 2015. Various species of wood are pyrolized in an inert atmosphere, the carbonaceous residue is saturated with calcium salts and finally reheated to obtain a highly porous crystallized material of much higher porosity than trabecular titanium or porous hard ceramic bone-substitutes; the inventors claim the wood based material will permit better penetration during bone growth and more flexion than metal or hard ceramic grafts [28]. Xenografts have given good results in dentistry, but scarce validation in orthopaedics.

Clinically available coral-based products are Interpore and Pro-osteon (Interpore International, Inc., Irvine, CA) as well as bovine derived products such as Bio-Oss (Geistlich Biomaterials, Geistlich, Switzerland), Osteograf-N (CeraMed Co., Denver, CO), and Endobon (Merck Co., Darmstadt, Germany). Doubts were argumented regard “zoonose” diseases transmitted from animals to humans, like BSE (Bovine spongiform Encephalopathy) or PERV (Porcine Endogenous Retroviruses) [29].

Results are contradictory with some authors reporting favourable data, but in the clinical practice xenografts are scarcely used [30–34].

Moreover poor results in hip surgery, with 25 % of pseudo infections complications, were recently reported [35].

The advantages are the easy availability, the osteoconductivity, the good mechanical properties and low costs.

## Ceramics

Generally ceramics bone substitutes are calcium based substitutes, a mix of HA (Hydroxyapatite) and TCP (Tricalcium.Phosphate), the amorphous phase of HA. HA is a relatively inert substance that is retained “in vivo*”* for prolonged periods of time, whereas the more porous TCP typically undergoes biodegradation within 6 weeks of its introduction into the area of bone formation. HA achieve very high mechanical strenght, while TCP has poor mechanical qualities. Generally the base is a biphasic calcium phosphate, which combine 40–60 % TCP with 60–40 % HA, that may yield a more physiological balance between mechanical support and bone resorption [36].

A level II and a level IV study found lesser pain, operating time, blood loss and complication in synthetic substitutes compared with iliac crest grafts [37]. Ceramics are widely known and are proved to be safe and effective in bone substitution. HA-TCP are now available in form of blocks, granules, and injectable kits. Macroporosity of about 100–400 µ and interconnected porosity are necessary for bone ingrowth. Depending on the concentration of HA and TCP the strength is variable between 10 and 60 MP that is very lower than cortical bone compression strength (150–200 MP), and this is one of the major limit of ceramic based biomaterials.

## Hydroxyapatite and tricalcium phosphate

**Fig. 4:**
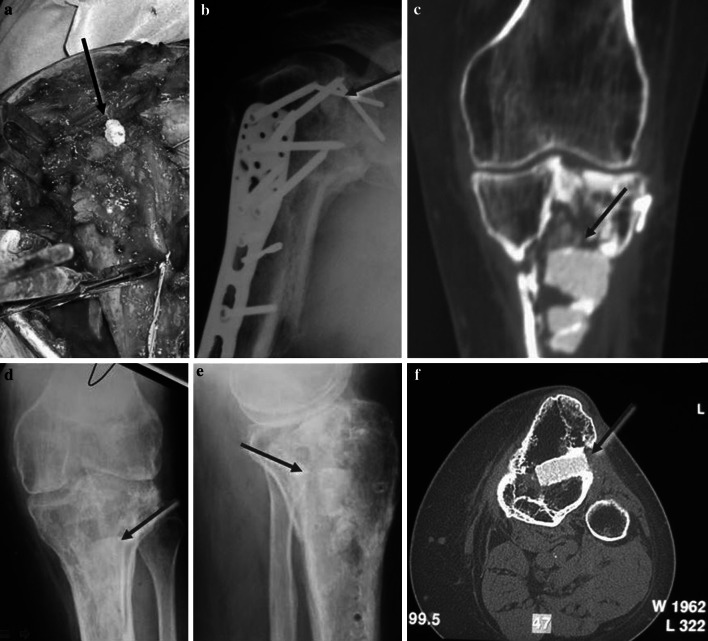
HA-TCP bone substitutes in proximal humeral and tibial traumatic bone loss. **a** Intraoperatory implant of the material in the proximal humerus. **b** X-ray control at 1 year show the substitute inside the humeral head. **c**–**f** X ray and CT scan at 3 year of follow up in the proximal tibia. The HA-TCP material resulted well osseointegrated, but without any sign of resorption or bone substitution

Hydroxyapatite [Ca_10_(PO_4_)_6_(OH)_2_] (HA) is the cristalline form of tricalcium phosaphate (TCP) and is the primary mineral component of teeth and bone. For the past 30 years, it has been popular in orthopaedic, craniofacial and orthognathic surgery, filling bony defects and smoothing contour irregularities. HA ceramics come in both naturally and synthetic forms. HA and TCP ceramics are manufactured in a variety of forms including granules and porous blocks (Fig. 4). TCP is more soluble than HA. Although HA accounts for nearly 70 % of the mineral content of teeth and bone, the occurring HA in the human body exists in a substituted form. Carbonate, silicates, and magnesium among other ions, may replace hydroxyl or phosphate groups of the apatite structure. Investigators have attempted to produce alginate [38], strontium [39], silicon [40], carbonate and magnesium [41–46] substituted synthetic HA in an effort to produce HA that more closely resembles the mineral content of native bone, enhancing bioactivity and osteoconduction (Biomimetic ceramic substitutes) [47]. Although there are few of products made of biomimetic HA in the clinical use at this time, the research is ongoing on this direction and biomimetic HA substitution will likely remain one of the most promising area of research.

## Calcium phosphate cements

**Fig. 5:**
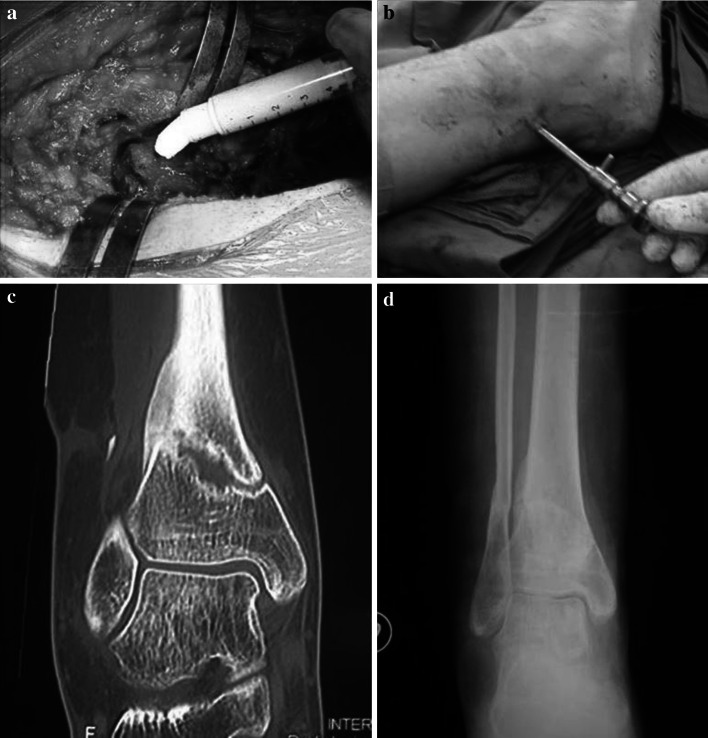
Injectable TCP cement bone substitutes: **a**–**b** injectable cements have the advantage to be mouldable and contourable to the bone loss in mininvasive or open surgery; **c** bone loss in a distal tibial open fracture delayed union (CT scan); **d** 1 year X-ray control, showed bone consolidation and osseointegration of the TCP cement

Calcium phosphate cements (CPC) are synthetic bone substitutes that were invented in 1986 by Chow and Brown, scientists at the American Dental Association [48]. The cements are a white powder, consisting of calcium phosphate, that when mixed with a liquid, forms a workable paste which can be shaped during surgery to fit the contours of bone loss. The cements harden within 20 min. The hardening reaction, which forms nanocrystalline hydroxyapatite (HA) is isothermic and occurs at physiologic pH so tissue damage does not occur during the setting reaction. CPCs were FDA approved for the treatment of non-load-bearing bone defects in 1996. HA is the primary inorganic component of natural bone which makes the hardened cement biocompatible and osteoconductive. Over time, CPCs are gradually resorbed and replaced with new bone. Because CPCs are brittle, they are used for non-load-bearing applications such as dental, cranio-facial and orthopaedic applications. CPCs have two significant advantages over pre-formed, sintered ceramics. First, the CPCs paste can be sculpted during surgery to fit the cavities. Second, the nanocrystalline hydroxyapatite structure of the CPC makes it osteoconductive causing it to be gradually resorbed and replaced with new bone. CPCs are injectable and were recently introduced in the clinical practice to adjuvate minimally invasive procedures (MIS) and tissue Sparing surgery (TSS) in order to reduce morbidity and costs (Fig. 5). The first cement that was introduced on the market in the late 1990s was the Norian, by Cupertino (Synthes-De-Puy), a self hardening carbonate HA, even known as “Dhallite” [49].

From then many other phosphate cements were proposed for clinical practice, like Bonesource (Stryker), Calcibone (Biomet), CrhonOs (Synthes), Hydroset (Synthes) [50], Sintlife (Finceramica) [47], KyphOs (Medronic).

Recently the research on CPC has focused on improving mechanical properties, making premixed cements, making the cement macroporous and seeding cells and growth factors into the cement.

## Calcium sulphate

**Fig. 6:**
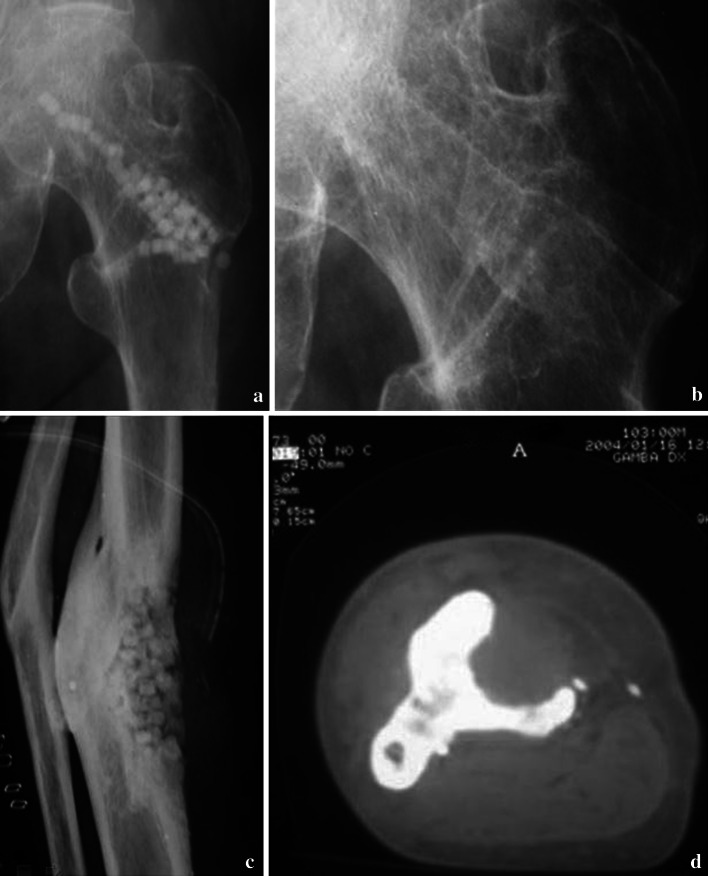
Calcium sulphate (CS): **a** Pellets fill the residual gap after DHS explant in a healed intertrochanteric fracture. **b** Two months after the CS was totally resorbed. **c** Antibiotic loaded CS pellets in a tibial osteomyelitis. **d** Three years CT scan control do not show any evidence of bone regeneration. No signs of CS were founded while the infection was healed

Calcium sulphate (CS), even known as “gypsum” or “Plaster of Paris”, was firstly implanted in humans as a void filler of tubercolous osteomyelitis by Dreesman in 1892 [51]. More recently it was reintroduced in the clinical practice as a bone substitute by Peltier in 1959, in a more pure and crystalline form [52–54] CS is resorbed variably within 6–8 weeks. Proponents of calcium sulfate claim that the pellets provide an effective gap filler, allow for vascular ingrowth, and resorb rapidly and completely, allowing for physiologic bone healing [55]. Apparently due to rapid graft resorption, the resulting calcium-rich fluid incites inflammation. First reports showed very promising results in vitro [56] and “in vivo*”*: Huff and Grisoni in the mouse [57], Cunningham in the sheep [58], Hadjiipavlou in the sheep [59], Turner in the dog [60], and also in humans [61, 62]. Recently many adverse or no effects were reported, mainly explained because of the too fast resorption and the production of a similar inflammatory reaction without bone formation (13–18 %) [63–67] (Fig. 6). Subsequently CS was proposed as a scaffold for demineralized bone matrix (DBM). The mixture with CS enhanced the clinical outcome more than calcium sulfate alone.

## Polymer-based bone graft substitutes

Polymers have physical, mechanical, and chemical properties completely different from the other bone substitutes. The polymers can be divided into natural polymers and synthetic polymers. These, in turn, can be divided further into degradable and nondegradable types.

One of the most important natural polymer in bone is collagen.

Cortoss is an injectable resin-based product with applications for load-bearing sites [68]. It consists of 33 % difunctional methacrylates that form a highly cross-linked 3-dimensional polymer, reinforced with 67 % radiopaque and bioactive glass ceramic particles. Initial results using Cortoss in vertebroplasty for osteoporotic and metastatic vertebral compression fractures were encouraging [69].

Degradable synthetic polymers (i.e., natural polymers) are resorbed by the body. The benefit is that they enhance healing without remaining foreign bodies. Degradable polymers such as polylactic acid and poly(lactic-co-glycolic acid) have been used as standalone devices and as extenders of autografts and allografts.

Most research has been directed to poly lactic acid (PLA), poly glycolic acid (PGA) and poly lactic-co-glycolide (PLGA) copolymers. Tissue Regeneration Therapeutics (Toronto, Canada) has developed a porous poly (lactic-co-glycolic acid) foam matrix by using a particulate-leaching process to induce porosity. It is currently marketed under the trade name OsteoScaf. Immix (Osteobiologics, Smith and Nephew, Memphis, Tennessee) and it is used as a graft extender [70].

The success of these has further led to the evaluation of aliphatic polyesters such as polyε-caprolactone (PCL). It appears as semicrystalline polyester and is highly processable as it is soluble in a wide range of organic solvents. The uncommon things of PCL is its high thermal stability, with decomposition temperature (Td) of 350 °C, whereas others aliphatic polyesters are between 235 and 255 °C [71]. In bone engineering, PCL can be categorized as a promising biocompatible and biodegradable polymer since it is being used to enhance bone ingrowth and regeneration in the treatment of bone defects [72, 73], however, PCL is poorly used due to the slow degradation time [74].

## Composite materials

### Composite of collagen and hydroxyapatite

Bone is mainly made of collagen (Col) and carbonate substituted hydroxyapatite (HA). Actually it is possible to obtain Col–HA by a self assembling process on a nanometric scale [75].

Thus, an implant manufactured from such components is likely to behave better than other bone substitutes made as monolithic devices. Indeed, both collagen type I and hydroxyapatite were found to enhance osteoblast differentiation [76], but combined together, they were shown to accelerate osteogenesis. A composite matrix when embedded with human-like osteoblast cells, showed better osteoconductive properties compared to monolithic HA and produced calcification of identical bone matrix [77, 78]. In addition, Col-HA composites proved to be biocompatible both in humans and in animals [77, 79]. Moreover these composites have some mechanical advantages. The ductile properties of collagen help to increase the poor fracture toughness of hydroxyapatites. The addition of a calcium/phosphate compound to collagen sheets gave higher stability, increased the resistance [80] and enhanced the mechanical ‘wet’ properties [81]. The direct comparison of other materials compared with Col–HA composites for bone substitutes have yet to be clearly investigated. However, increasing the biomimetic properties of an implant may reduce the problems of bacterial infections associated with inserting a foreign body [82]. Evidence of the biological advantage compared to artificial polymeric scaffolds have been further demonstrated in cartilage regeneration [83]. The addition of collagen to a ceramic structure can provide other additional advantages to surgical applications: shape control, spatial adaptation, increased particle and defect wall adhesion, and the capability to favor clot formation and stabilization [79].

Healos (DePuy Orthopaedics, Inc, Warsaw, Ind) is a natural polymer-based product, a polymer-ceramic composite consisting of collagen fibers coated with hydroxyapatite and indicated for spinal fusions [84].

In summary therefore, combining both collagen and hydroxyapatite should provide an advantage over other materials for use in bone tissue repair [85]. Further clinical studies are required to validate its effectiveness.

## Growth factors

### Demineralized bone matrix

**Fig. 7:**
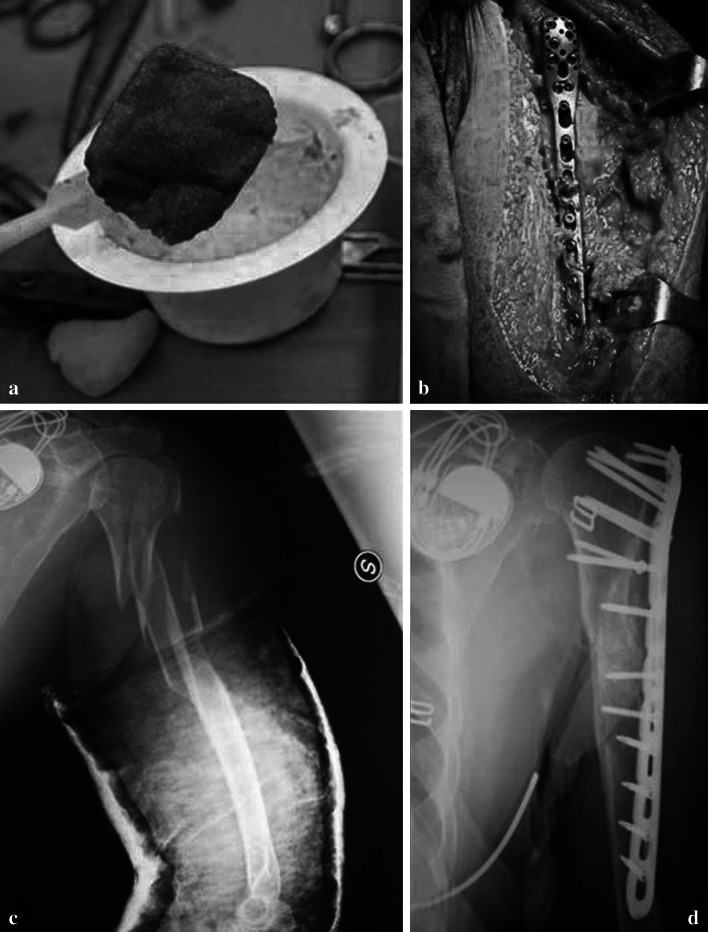
Demineralized Bone Matrix (DBM): **a**–**c** Complex proximal humeral fracture treated by ORIF, DBM and calcium sulphate (Allomatrix-Wright); **d** One year follow up demonstrate good consolidation of the fracture

The aseptical processing of banked donor bone to produce human demineralized bone matrix (DBM), was first described in 1975 [86] and introduced in the orthopedic and periodontal practice since the early 1980s. The decalcified bone(residual calcium <5 %) is mainly represented by the collagen matrix that is supposed to replicate the tridimensional architecture of bone, hence facilitating and guiding host cell invasion, growth and differentiation [87]. Moreover DBM should retaining bone-inducing growth factors, such as bone morphogenetic proteins (BMP), insulin growth factor (IGF), transforming growth factor (TGF), fibroblast growth factor (FGF), able to stimulate activation and migration of osteogenic stem cells and progenitor cells, and to induce revascularization. Nonetheless, DBM lacks of any mechanical strength, hence are used exclusively for filling purposes and normally are associated with carriers like glycerol (Grafton-Osteotech), calcium sulphate (Allomatrix-Whright), hyaluronic acid (DBX-Synthes), porcine collagen (Osteofil- Medtronics-Sofamor Danek), carbossimetilcellulose (Dynagraft- GenSci Regeneration Sciences) (Fig. 7). Despite a copious amount of literature data reports the effectiveness of DBM, mostly in preclinical studies [20], as a safe and effective bone grafting material [88], there is still limitate evidence produced in Level 1-2 studies, to support the use of DBM as a stand-alone bone substitute [89].

Moreover, concerns were raised about the extreme variability in BMP 2 and BMP 7 content in different commercially available DBM lots, which may be due to the absence of standardized processes for production along with donor-related issues [89, 90].

### Platelet rich plasma (PRP)

**Fig. 8:**
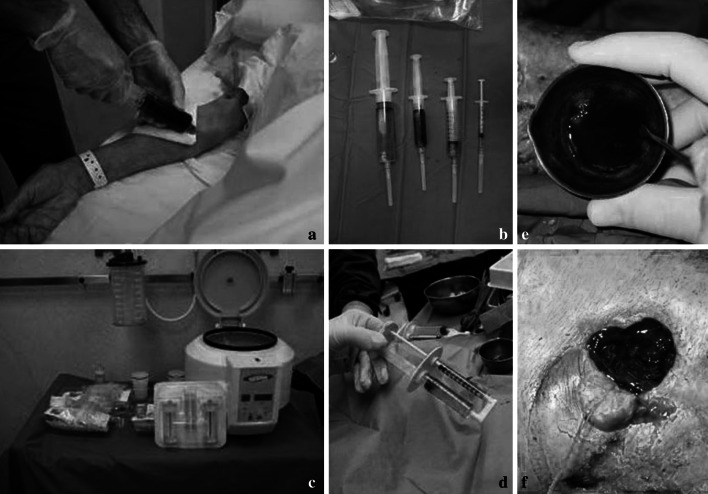
Platelet Rich Plasma (PRP): **a**–**d** autologous blood is obtained in the operating room. After centrifugation the different components are differentiate. **e**, **f** A platelet concentrate is obtained for injection or deposition into the bone gap or wound

Blood platelets are easily collected from blood and represent a valuable source of growth factors, such as the platelet derived growth factor, the insulin-like growth factors, and the transforming growth factors [91–93]. Platelet-rich plasma (PRP) is easily obtained by concentration of autologous blood platelet through gradient density centrifugation (Fig. 8). PRP proved to exert chemotactic and mitogenic properties for osteoblast and fibroblast cells in vitro, to stimulate fibroblast hyaluronate synthesis, a pre requisite for the formation of the extracellular matrix, thereby enhancing bone formation [94–98].

Unfortunately, diverse clinical studies so far reported unsuccessful results in spine surgery, with a decreased incidence of spine fusions (15–19 %), even when used in combination with bone marrow cells [99]. This failure was believed to be due to a paradoxical inhibitory effect on BMP 2 at high concentrations [100–102]. Indeed, currently PRP is not validated as a stand-alone bone substitute but is rather considered a co-growth factor for bone healing.

### Bone morphogenetic proteins

Originally identified and named after their ability to induce ectopic bone formation [103], bone morphogenetic proteins (BMPs) represent a wide and heterogeneous family of highly conserved secreted proteins, within the transforming growth factor-β superfamily, deeply involved in the skeletogenic process [104, 105].

Selected BMPs appear to promote in vitro bone formation by: inducing the differentiation of pluripotent mesenchimal cells towards the chondrogenic and osteogenic lineages, stimulating angiogenesis and alkaline phosphatase activity [94]. The osteogenic/osteoinductive potential of the BMPs was strongly validated in both preclinical and clinical studies, generally reporting performance that were comparable to autogenous cancellous bone, with fusion rates between 80 and 99 % [106–112].

To date, only the use of recombinant human BMP2 and BMP7 has been approved both in Europe and the United States for selected clinical applications: BMP 2 with a collagen carrier (INFUSE, Medtronic Sofamor Danek, Minneapolis) for lumbar vertebral interbody fusion and BMP 7 (OP-1, Stryker, Kalamazoo, Michigan) for tibial non union, in patients who underwent previous unsuccesfull treatments. Dark and lights persist also due to the variable needed dosage, which may be patient- and site-dependent, and to the still high costs, which makes their use prohibitive in most settings. In addition, BMPs showed adverse effect in cervical spine and are hence contraindicated in this application [113].

## Emerging strategies for bone substitution

### Biomimetic and smart materials in bone tissue engineering

The challenge to tissue engineers is to design and develop temporary bone scaffolds which deliver bioactive molecules and drugs or cells to the injury site and hence extend its biological functionality (accelerate healing and tissue regeneration while simultaneously preventing pathology). Although mimicking the geometric architecture of bone in a synthetic scaffold has been shown to promote favorable cellular activity, the overall capacity for a scaffold to direct cell behavior can be substantially improved through the controlled delivery of bio specific cues [114–119]. Administration of growth factors and other bioactive molecules to promote bone formation and repair has achieved promising results in several preclinical and clinical models [120–125]. A variety of administration methods have been investigated including: bolus injection, surface adsorbed protein release, osmotic pumps, and controlled release from biodegradable scaffolds. The efficacy of the delivery vehicle relies on its ability to provide the appropriate dose over the appropriate therapeutic time. Ideally, the presentation of bioactive molecules or drugs must be finely tuned to dynamically match the physiological needs of the tissue as it regenerates. Because of the hydrolytically unstable linkages in their backbone and tunable biodegradation rate, polymers have demonstrated to be effective. Ceramic materials have also demonstrated the ability to biodegrade and release bioactive molecules at a controlled rate [126–130]. Natural polymers such as collagen, fibrin, alginate, gelatin, and GAGs have also been extensively investigated as drug delivery vehicles in bone tissue engineering. These natural polymers have distinct advantages due to their inherent biocompatibility and bioactivity but lack the mechanical properties required for load bearing applications, may have inappropriate (fixed) degradation rates, are difficult to harvest and sterilize, and may induce an immunogenic response. Bioactive molecules can be covalently bound to polymers or physically entrapped inside a polymer matrix [131, 132]. In either case, the molecule is released as the polymer degrades in the physiological environment.

Aliphatic polyesters such as poly(lactic-acid)(PLA), poly(glycolic-acid)(PGA), and poly(caprolactone) (PCL), and their copolymers are the most commonly utilized polymers in bone tissue engineering [133–135]. Both PGA and PLA scaffolds has been investigated as a slow-delivery carrier for growth factors in several “in vitro” and “in vivo” studies, and demonstrated the ability to promote healing and osseointegration compared with control scaffolds [136–138]. Researchers combine multiple polymers in a chemical process called copolymerization to gain more control over the degradation rate, hydrophobicity, crystallinity. Copolymerization is analogous to the design of composite materials where multiple constituents are combined resulting in a new material with desirable properties from each constituent. Undoubtedly, the most commonly utilized copolymer for bioactive molecule encapsulation and release for bone tissue engineering is the copolymer poly(lactic acid-*co*-glycolic acid) (PLGA). Several researchers have utilized this well-characterized copolymer for encapsulation and release of a wide variety of bioactive molecules and drugs including TGF-β, BMPs, IGFs, VEGF, NGF, DNA, vancomycin, gentamycin, cisplatin, and others [139–146]. However, although PLGA has shown to be promisinge in bone scaffold applications, its clinical utility is limited due to its relatively poor mechanical properties (specifically Young’s Modulus) compared with cancellous bone, and therefore must be combined with other materials to enhance its mechanical properties [147].

Many synthetic bone scaffolds rely on the delivery of single factors, which may partially explain the limited clinical utility of many current approaches [140]. Therefore, researchers have been investigating techniques to encapsulate and release multiple bioactive molecules in a highly controlled spatial and temporal manner. Research has shown that this method significantly enhances tissue regeneration compared with the controlled release of single biological cues [148–150]. The technology of incorporating multiple chemical effectors and controlling their spatial and temporal release is a very promising strategy, but is still experimental and has not yet demonstrated widespread preclinical or clinical utility to date.

The failure to identify either a single material or growth factor as the panacea for bone regeneration, or a biological scaffold that will promote integration and vascularization, has led to an increased interest in optimizing biomaterials to promote specific cell-biomaterial interactions. For example, Arg–Gly–Asp (RGD) sequence peptides (involved in integrin-mediated cell adhesion) can be incorporated onto the scaffold surface to enhance cell adhesion and spreading [151]. Yang et al. [152] have demonstrated the potential to promote human osteoprogenitor differentiation on RGD-coupled biodegradeable scaffolds.

More recently drug delivery techniques such as entrapment within a hydrogel matrix allowing growth factors to be released in a controlled fashion from the scaffold to aid the regenerating tissue have been applied [153–156]. Such approaches are appealing as they avoid the use of solvents, and high temperatures (and therefore protein degradation) and subsequent release of the growth factor is controlled, in response to environmental stimuli. This strategy has been employed in bone tissue engineering, where rhBMP-2 [157] basic fibroblast growth factor [155] and vascular endothelial growth factor [156] have all been successfully incorporated into a hydrogel prior to in vivo implantation. Supercritical fluid technology has evolved as a promising approach in the development of porous biodegradable scaffolds for tissue engineering [158]. The absence of solvents and thermal processing makes this an attractive approach to growth factor incorporation and Howdle and colleagues have demonstrated high protein (ribonuclease) loading into foamed PLA scaffolds which retained full activity on subsequent release from the PLA over 3 months [153, 159]. This technology could provide a simple one-step process to the difficulties of incorporating growth factors and/or guest particles (such as hydroxyapatite) into a controlled release delivery system.

New strategies works to encapsulate and release drugs which prevent pathologies that can occur post implantation of a synthetic scaffold. A wide variety of drugs have been encapsulated and released from biodegradable polymer scaffolds including antibiotics, DNA, RNA, cathepsin inhibitors, chitin, chemotherapeutics, bisphosphonates, statins, sodium fluoride, dihydropyridine, and many others [160–164]. Researchers are aggressively pursuing strategies to deliver antibiotics locally to the site of injury/surgery. The most common biodegradable polymer/antibiotic combination is PLGA scaffolds loaded with antibiotics such as ciprofloxin, gentamycin, and vancomycin [165, 166]. PLGA scaffolds have demonstrated successful sustained local delivery of these antibiotics for up to 20 or more days in vitro and in preclinical animal models [141, 142, 167–169]. Although local delivery of antibiotics has a very promising outlook, there remains a number of challenges (such as antibiotic stability within the scaffold and antibiotic deactivation during fabrication), which need to be addressed before clinical trials can begin [170].

### Cell-based and gene therapy

The challenges offered by current bone grafting techniques have been driving the intensive research efforts spent during the last decades to develop new approaches and technologies. To this aim, cell-based gene therapy has attracted great interest from the scientific community, representing a tentative approach to achieve bone substitution [171]. In gene therapy approaches, cells are used as bioactive vehicles delivering osteoinductive genes locally to achieve bone regeneration. Different molecules have been tested to this aim, mainly represented by genes belonging to the BMP family and related cascade [172]. Indeed, genetically engineered cells are believed to maintain physiologic doses of a gene product for a sustained period once inoculated into the selected anatomical site, facilitating an efficient bone healing [173]. In addition, the overwhelming amount of data that have been clarifying the whole molecular scenario orchestrating osteogenesis and bone healing, provided new osteoinductive molecules to be tested as potential therapeutics [174]. Cell-based gene therapy approaches based on engineered-osteoinductive cells allowed achieving the most convincing results in terms of bone healing in animal models [172, 174, 175]. Though, a number of safety issues currently limit the use of genetic engineering procedures, based on viral and nonviral vectors, in the clinical setting. Thus, strictly-named gene therapy approaches for bone regeneration ceased to represent suitable for translational purposes.

Cell-based approaches are mainly based on mesenchymal stem cells (MSCs), that have been widely employed, in conjunction with appropriate osteoinductive scaffolds, and considered the most effective bioactive bone substitutes “in vivo” [172, 176, 177].

MSCs are multipotent stem cells that are capable of extensive self-renewal, plasticity and multilineage potential [178, 179]. These cells resides in the stroma of bone marrow and other organs and tissues (fat, muscle, skin, synovial membrane, tendons lung, etc.); hence they are also named “stromal stem cells” [180]. The great advantages of MSCs reside in the ease of isolation and “ex vivo*”* expansion, preserving their plasticity and self-renewal potential [181]. Upon appropriate in vitro induction, MSCs can be differentiated along the osteogenic lineage. MSC derived from bone marrow showed a high potential for osteogenic differentiation, which has been exploited for cell-based therapy of congenital bone disorders [182–184]. The proposed use of MSCs in orthopedic surgery comes also from their immunomodulatory properties, that make them potentially suitable for allogenic transplantation [185].

It is noteworthy that naïve undifferentiated MSCs are prone to environment-induced lineage commitment [186] meaning that they can undergo spontaneous osteogenic differentiation upon “in vivo*”* inoculation into a damaged bone [187, 176]. This feature may suggest their safe use as it does not imply any kind of “ex vivo*”* osteogenic induction prior to “in vivo*”* inoculation. Nonetheless, the production of clinical-grade MSCs, requires dedicated cell factories for their “ex vivo*”* large scale culture amplification; these are GMP-proof facilities that need to comply to the same regulations required for the drug manufacturing industries, as culture-amplified cells, according to European tissue banking rules, are considered as medicinal products [21, 188].

Few active clinical trials are currently ongoing that exploit MSC-based treatment as bone regenerative strategies (www.clinicaltrials.gov) [189].

## Conclusions

Bone loss persists to be an important challenge in surgery, and many alternatives are available. Despite the improvement of research, human bone grafts persist to be the most effective bone substitutes to replace bone loss. Alternatives to bone grafts lacks of one or more of the concepts of the “Diamond theory” of Giannoudis: osteogenic cells and vascularization, mechanical stability, growth factors, osteoconductive scaffolds (in combination with growths factors), that are a prerequisite for bone healing [190]. Moreover most alternatives are expensive and not validated by EBM, thus being scarcely recommendable for clinical use. Actually, ceramics substitutes are the best for safeness, effectiveness and costs. BMP 2 and BMP 7 are EBM validated, for specific use, but costs are elevated. Other techniques or alternatives are expensive, and not validated, thus needing standard randomized clinical trials prior to be approved for routinely clinical use.
